# Romiplostim for Prevention of Severe Chemotherapy-Induced Thrombocytopenia in Lymphoma Patients—Phase I Study

**DOI:** 10.3390/cancers18020188

**Published:** 2026-01-06

**Authors:** Erel Joffe, Zachary Epstein-Peterson, Lorenzo Falchi, Ariela Noy, Andrew D. Zelenetz, Collette Owens, Leah Gilbert, Gilles Salles, Gerald A. Soff

**Affiliations:** 1 Division of Hematologic Malignancies, Memorial Sloan Kettering Cancer Center, New York, NY 10065, USA; 2 Faculty of Medicine, Hematology, Tel Aviv University, Tel Aviv 6997801, Israel; 3 Weill Cornell Medical College, New York, NY 10065, USA; 4 Division of Hematology, Sylvester Comprehensive Cancer Center, University of Miami Health System, Miami, FL 33136, USA

**Keywords:** severe chemotherapy induced thrombocytopenia, lymphoma, romiplostim

## Abstract

**Background/Objectives:** Intensive chemotherapy is the cornerstone of lymphoma treatment but often leads to severe chemotherapy-induced thrombocytopenia (sCIT), resulting in treatment delays, reduced dose intensity, and the need for transfusions. While granulocyte colony-stimulating factors (G-CSFs) are commonly used to manage neutropenia, the use of thrombopoietic growth factors has not been adequately studied. **Methods:** This phase I dose-finding study evaluated the use of weekly romiplostim as prophylaxis for recurrent sCIT in patients undergoing chemotherapy for lymphoma. Eligible patients were those treated with a 21-day chemotherapy cycle who previously experienced sCIT, thus serving as their own “controls”. sCIT was defined as one of the following: (A) a platelet count (PLT) <50 × 10^9^/L on day 1 of the subsequent cycle, leading to delay or dose reduction in chemotherapy, or (B) grade 4 thrombocytopenia (<25 × 10^9^/L) and/or (C) platelet transfusion for bleeding. The primary endpoints were the incidence of sCIT and the rate of romiplostim-associated-adverse-events, with thromboembolic complications being an event of special interest. **Results:** Nine patients with sCIT requiring a PLT transfusion on the prior treatment cycle were treated across three dose schedules. The phase 2 recommended schedule was defined as a starting dose of 3–5 mcg/kg based on the baseline PLT count, with weekly adjustments for counts <150 × 10^9^/L and >450 × 10^9^/L. Romiplostim prevented recurrent grade 4 thrombocytopenia in 47% of the chemotherapy cycles and averted recurrent transfusion in 65% of cycles. Notably, low starting doses, as used in solid malignancies, were insufficient, leading to recurrent thrombocytopenia. **Conclusions:** Romiplostim was well-tolerated, with no thromboembolic events, and allowed most patients to complete their chemotherapy on schedule at full dose intensity.

## Simple Summary

Lymphoma patients receiving intensive chemotherapy frequently develop severe chemotherapy-induced thrombocytopenia, characterized by critically low platelets that increase bleeding risk, necessitate platelet transfusions, and often force treatment delays or dose reductions. While pharmacologic growth factors are routinely used to manage chemotherapy-induced neutropenia, thrombopoietic agents remain inadequately studied. This phase I study investigated whether secondary prophylaxis with weekly romiplostim administration could prevent recurrent severe thrombocytopenia in lymphoma patients undergoing chemotherapy who had already experienced profound platelet drops requiring transfusions in prior cycles. Nine patients were enrolled across three dose schedules to establish a recommended phase 2 dose schedule. Romiplostim effectively prevented grade 4 thrombocytopenia in nearly half of the chemotherapy cycles and substantially reduced platelet transfusion requirements in this high-risk population. The agent was well-tolerated without thromboembolic complications, enabling most patients to maintain their planned chemotherapy schedule at full dose intensity. These findings establish a dosing framework and suggest that secondary prophylaxis with romiplostim may represent a viable strategy to optimize chemotherapy delivery in lymphoma patients.

## 1. Introduction

Severe chemotherapy-induced thrombocytopenia (sCIT) represents a significant clinical challenge in the management of high-grade lymphoma. Despite the frequency and adverse impact of sCIT, no validated treatments currently exist beyond chemotherapy dose reduction or delay. sCIT is most notable with the use of intensive frontline regimens for mantle cell lymphoma (MCL) and advanced-stage Hodgkin’s lymphoma (HD) where up to 50% of patients may experience a grade 4 thrombocytopenia (platelet count ≤ 25 × 10^9^/L), often necessitating frequent platelet transfusions [1,2]. In intensive chemotherapy regimens used in the treatment of relapsed or refractory (R/R) lymphoma or chronic lymphocytic leukemia, 30–60% of patients develop severe thrombocytopenic complications [3,4,5]. These patients require constant monitoring and repeated platelets transfusions, face the risk of severe hemorrhagic complications, and may be unable to complete the prescribed regimen, thus compromising treatment efficacy.

While granulocyte-stimulating factors (G-CSFs) have revolutionized the management of neutropenia, thrombopoietin receptor agonists (TPO-RAs) remain comparatively underexplored in the setting of sCIT. Use of the TPO-RA romiplostim to prevent sCIT, defined as a platelet count <100 × 10^9^/L persisting for at least 3 weeks from the date of last chemotherapy administration or necessitating a greater than one week delay in the planned administration of further chemotherapy, was evaluated in a prospective study of 52 patients with solid tumors [6]. Eighty-five percent of all patients treated with romiplostim achieved corrected platelet counts (>100 × 10^9^/L) within 3 weeks. A subsequent retrospective multi-center trial of romiplostim in 173 patients with sCIT demonstrated that 71% achieved a response (defined as a PLT increase to ≥75 × 10^9^/L and ≥30 × 10^9^/L above baseline), 79% avoided chemotherapy dose reductions/delays and 89% avoided platelet transfusions [7]. Notably, of the 20 of the patients in that study with non-myeloid hematological malignancies (lymphoma or multiple myeloma), only 35% demonstrated an adequate platelet response to romiplostim, while nearly half of the patients required chemotherapy dose reductions [7]. Following these studies, the National Comprehensive Cancer Network (NCCN) added a level 2A recommendation for the use of romiplostim for the treatment of platelets <100 × 10^9^/L for ≥3–4 weeks following the last chemotherapy administration and/or following delays in chemotherapy initiation related to thrombocytopenia [8].

Notably, the traditional definition of sCIT stemming from the above research in solid malignancies may not be applicable for regimens used in the treatment of hematological malignancies. sCIT of hemato-oncological regimens tends to be much more profound, meeting the Common Terminology Criteria for Adverse Events (CTCAE) for grade 4 toxicity (PLT < 25 × 10^9^/L) in 40–60% of patients, with a considerably increased risk of bleeding and often requiring platelet transfusions (PLT < 10–20 × 10^9^/L, varying by specific institutional guidelines) [3]. Furthermore, these regimens tend to be limited to few treatment cycles where maintaining PLT counts over long periods of time is not as important as timely PLT count recovery, which is necessary for maintaining treatment schedule and dose intensity [9,10]. Thus, rather than the traditional definition of sCIT (platelet count <100 × 10^9^/L persisting for at least 3 weeks), defining sCIT in the setting of intensive salvage regimens in lymphoma may be more clinically meaningful if it is focused on the occurrence of profound grade 4 thrombocytopenia or the need for platelet transfusions.

Prior to the introduction of thrombopoietin TPO-RAs, a small-phase I/II randomized placebo-controlled study demonstrated that the use of pegylated recombinant human megakaryocyte growth and development factor (PEG-rHuMGDF) in patients with R/R DLBCL treated with high-dose chemotherapy was associated with a considerably lower rate of grade 4 thrombocytopenia and platelet transfusion requirements [11]. Chemotherapy dose intensity was improved with the use of PEG-rHuMGDF support, and there was a trend toward improved overall survival in these patients. Unfortunately, use of PEG-rHuMGDF was associated with the development of auto-antibodies against thrombopoietin leading to discontinuation of this agent [12].

Our current study aimed to evaluate weekly romiplostim administration in the prevention of recurrent sCIT in patients treated with chemotherapy for lymphoid malignancies.

## 2. Materials and Methods

This was a single-center, open-label phase I study evaluating the use of romiplostim for the prevention of recurrent sCIT in patients with lymphoma. Patients were eligible if they were treated on a chemotherapy regimen with a 21-day cycle and had experienced a sCIT, defined as one of the following: (A) a platelet count (PLT) <50 × 10^9^/L on day 1 of the subsequent cycle, leading to delay or dose reduction in chemotherapy, (B) grade 4 thrombocytopenia (<25 × 10^9^/L) and/or (C) platelet transfusion for bleeding in their prior cycle of chemotherapy. Patients had to have at least one more planned cycle of treatment. Patients were excluded if they had a prior allogeneic hematopoietic stem cell transplant; a history of venous thrombotic events requiring anticoagulation but an inability to tolerate treatment; a history of a symptomatic arterial thrombotic event within the prior 4 months; or thrombocytopenia related to pre-existing immune thrombocytopenic purpura (ITP). Patients were to be withdrawn from the study if they required a dose reduction in chemotherapy on subsequent cycles. Stem cell or T cell collection was not allowed during the first cycle on protocol but was allowed after C2D14 administration (corresponding to the third cycle or later of the chemotherapeutic regimen), to avoid fluctuations in PLT counts following pheresis. This study was approved by the Memorial Sloan Kettering Cancer Center institutional review board (IRB #20-492) and registered at ClinicalTrials.gov identifier NCT04673266, registered 14 December 2020. Research data are not shared.

**Figure 1:**
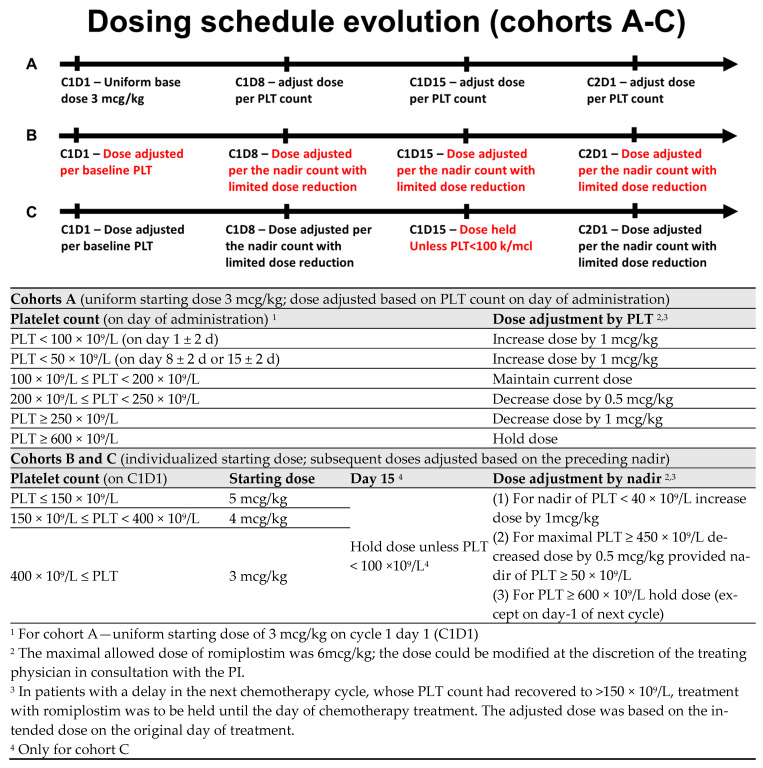
Dosing schedules.

Romiplostim was administered weekly from the beginning of the chemotherapy cycle (a window of +2 days). There were three dosing cohorts in this study. In cohort A, all patients started with a fixed dose of romiplostim of 3 mcg/kg. Subsequent weekly doses were then titrated according to platelet counts on the day of weekly romiplostim administration (Figure 1). For PLT ≥ 600 × 10^9^/L, treatment was held. After observing multiple sCIT events on days 8 and 15 (seemingly associated with a low starting dose), as well as recurrent sCIT events following per-protocol dose reductions; the protocol was amended to include increased doses of romiplostim (3–5 mcg/kg) on the first day of treatment, adjusted for baseline PLT count, as well as a more stringent criterion for dose reduction (cohort B; subjects 7 and 8). Further, the dose was then to be adjusted and maintained based on the observed nadir rather than the PLT count on the day of administration (Figure 1). After observing spikes in PLT to above 600 × 10^9^/L on C2D1 in cohort B, the schedule was further amended to omit the dose on day 15 provided that the PLT count was ≥100 × 10^9^/L (Cohort C; subject 9 and 10; Figure 1). For all the cohorts, the maximum dose of romiplostim was capped at 6 mcg/kg.

The primary endpoint was a composite of any of the following during the first cycle on the study drug: (1) indication for dose delay, defined as PLT < 50 × 10^9^/L, on the planned first day of the subsequent cycle; (2) grade 4 thrombocytopenia, defined as PLT < 25 × 10^9^/L, at any point during the treatment cycle; or (3) platelet transfusion for thrombocytopenia or bleeding at any point during the cycle.

Secondary endpoints were (1) to assess the safety and tolerability of romiplostim in patients with lymphoma and sCIT secondary to chemotherapy, with thrombotic events defined as adverse events of special interest, and (2) to evaluate the composite endpoint during the second cycle of treatment.

## 3. Results

Nine patients enrolled in the study were treated with 17 treatment cycles of chemotherapy (with all but one completing 2 cycles of trial treatment). Prior to enrollment, in the qualifying chemotherapy cycle, all patients had experienced grade 4 sCIT and all required at least one platelet transfusion (Table S1). All patients had recovered counts to meet the criteria for chemotherapy administration by day 1 of the subsequent chemotherapy cycle (C1D1 of the trial). All patients received prophylactic pegylated gCSF as part of their regimen. Table 1 presents the baseline characteristics of the study participants.

Of the 17 cycles with romiplostim prophylaxis (nine in cohort A; eight in cohort B + C; Table 2), grade 4 sCIT was seen in nine cycles (53%), and transfusions were necessary in six (35%). Notably, all but two grade 4 sCIT events were observed in patients treated on cohort A with an initial dose of 3 mcg/kg and subsequent dose reductions. Of the four patients on cohort B and C, only one patient experienced grade 4 sCIT (25%).

**Figure 2:**
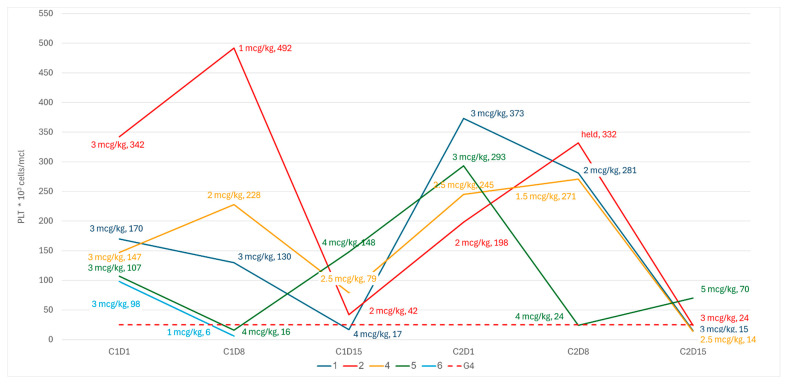
PLT kinetics and romiplostim dose on weekly dose adjustments.

All the patients in cohort A experienced at least one episode of grade 4 sCIT, either during the first cycle of treatment or following per-protocol dose reductions during cycle 2 (Figure 2). In two patients (pt-5 and pt-6), we observed an early grade 4 thrombocytopenia during the first week, before any dose adjustment could be made. In two patients (pt-2 and pt-4), there was a spike in PLT during the first week, leading to a per-protocol romiplostim dose reduction that was followed by a steep drop in PLT due to C1D15. All patients in cohort A showed dose reductions in romiplostim during the second cycle, resulting in grade 4 sCIT.

**Figure 3:**
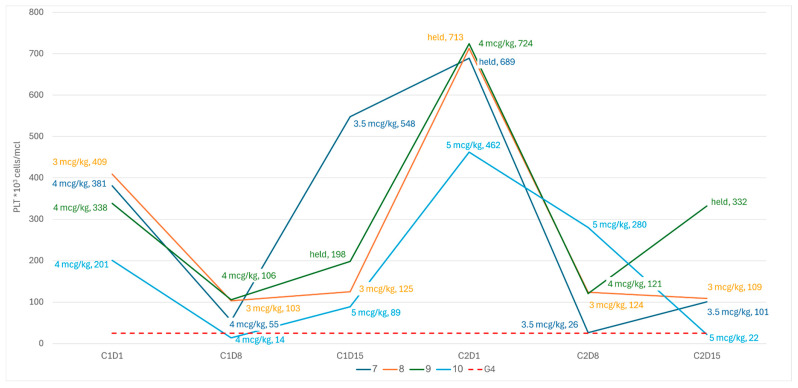
PLT kinetics and romiplostim dose revised dosing schedule.

Following these observations, the dosing schedule was amended to start with higher doses on C1D1, adjusted to the platelet count, with more stringent criteria for dose reductions based on the nadir count during prior weeks. Following the amendment, although neither of the next two patients had experienced a grade 4 sCIT, both demonstrated a spike in the PLT count to ≥650 × 10^9^/L due to C2D1, leading to the maintenance of the treatment dose (Figure 3). Subsequently, both patients demonstrated a steep decline in platelets due to C2D8 (nearly meeting the criteria for grade 4 thrombocytopenia in pt-7), and so the dosing schedule was amended to omit the treatment dose on day 15 of the cycle, provided that the patient was clearly recovering based on the nadir of the counts (i.e., PLT > 100 × 10^9^/L). Two additional patients were treated in accordance with the final revised schedule (Figure 3).

There were no thromboembolic complications noted, nor any other romiplostim-related adverse events. There were no bleeding events. All observed adverse events were deemed unrelated to romiplostim and attributed to chemotherapy.

## 4. Discussion

Intensive chemotherapy regimens are a cornerstone of lymphoma treatment but are frequently complicated by severe treatment-related cytopenia. While prophylactic growth factors are routinely used to manage neutropenia, thrombopoietic growth factors have not been adequately studied in lymphoma.

This study evaluated the use of romiplostim in patients experiencing grade 4 thrombocytopenia during intensive chemotherapy regimens for lymphoma. Our primary goal of this phase 1 study was to establish a dosing schedule tailored to the kinetics of thrombocytopenia in lymphoma. Romiplostim successfully prevented recurrent grade 4 thrombocytopenia in 47% (8/17) of intensive chemotherapy cycles and offset the need for transfusions in 65% (11/17). Notably, low starting doses, as used in solid malignancies, were insufficient, leading to recurrent thrombocytopenia. Per the revised dosing schedule, higher starting doses were associated with fewer episodes of sCIT (1/4 patients on cohorts B and C). These small numbers are far from definitive but provide guidance for a subsequent study.

Additionally, premature romiplostim dose reduction before full recovery from the thrombocytopenic nadir (Day 15 of study cycle) was linked to subsequent grade 4 thrombocytopenia. Avoiding mid-cycle dose reductions, as implemented in the revised schedule, might have prevented thrombocytopenia in three cases in cohort A on cycle 2.

Importantly, romiplostim was well tolerated, with no added toxicity, and without thromboembolic complications, and allowed all but one patient (who withdrew consent) to complete their planned chemotherapy on schedule and at full dose intensity.

To put our observations in context, prophylactic use of G-CSF in malignant lymphoma has not been a definitive treatment for neutropenia; rather, it has decreased the risk of severe complications (grade 4 neutropenia, neutropenic fever or infection) by 30–40% [13]. Similarly to the PLT count kinetics we observed following the administration of romiplostim, G-CSF administration has been associated with an early spike in neutrophil count, at times mimicking a leukemoid reaction, that has not been associated with serious clinical implications. In some of the patients in this study, a transient spiking in PLT counts up to 600–800 × 10^9^/L was observed. These values led to frequent dose reduction driven by a concern for thromboembolic events. As with prior studies, we did not observe any thromboembolic events [6,7,11,14,15]. Further, as with G-CSF, where the neutrophil spike often precedes the nadir of the neutropenia, thrombocytosis often preceded the development of thrombocytopenia, particularly in cases where the dose of romiplostim was either reduced or maintained [16].

Primary prophylaxis with G-CSF has been advocated for all patients undergoing treatments with a 20% or higher risk of febrile neutropenia and should be considered for regimens associated with a 10–20% risk [8]. Secondary prophylaxis is advised for all patients with a prior event of neutropenic fever. sCIT complicates 30–50% of treatments administered for relapsed or refractory lymphomas, as well as common regimens used in the frontline treatment of Hodgkin’s, mantle, primary CNS, and Burkitt’s lymphoma [1,3,17,18,19]. It is increasingly recognized as a significant concern in newer regimens incorporating novel therapies [2,4,5]. This represents a critical unmet need, and should further studies validate the safety and efficacy of TPO-RAs in this context, their prophylactic use would align with current G-CSF guidelines.

The main limitation of this study is the modest sample size. Due to funding constraints, this phase I, investigator-initiated study did not allow for the evaluation of additional dosing schedules nor for the expansion to a phase II stage. Furthermore, it should be noted that the nadir of PLT counts may differ between cycles in the same patient. On the other hand, the statistical power of this study is enhanced by the within-subject repeated-measures design, wherein each patient functioned as their own control, having experienced sCIT necessitating platelet transfusion during the preceding treatment cycle. This methodological approach confers sufficient statistical power to draw preliminary inferences. The final dosing schedule used for cohort C may be a reasonable recommendation as a phase II dosing schedule.

## 5. Conclusions

Secondary prophylaxis of sCIT with a weekly dosing schedule adjusted to the baseline and nadir of platelet count seems safe and efficacious in preventing recurrent events of sCIT.

## Figures and Tables

**Table 1:** Baseline patient characteristics at time of enrollment.

ID	Dose Delay	PLT Transfusion	Age	Histology	Disease Status	Prior Lines	Stage	BM	Regimen	Cohort	Outcome
**1**	0	2	57	DLBCL	de novo	0	4	Yes	[RCHOP]-RICE	A	CR
**2**	0	1	69	DLBCL	de novo	0	4	Yes	[RCHOP]-RICE	A	CR
**3**	Screen Failure										
**4**	0	2	56	DLBCL	relapsed	1	4	No	RICE	A	CR; continued to ASCT—NED since
**5**	0	2	75	AITL/tDLBCL	relapsed	1	4	No	RGemOx	A	CR; continued to ASCT—NED since
**6**	0	1	64	DLBCL	relapsed	2	4	Yes	RDHAOx	A	Withdrew consent—due to unrelated AE
**7**	0	2	75	DLBCL	relapsed	2	2	No	RDHAOx	B	CR; continued to ASCT—NED since
**8**	0	1	37	DLBCL	relapsed	1	2	No	RDHAOx	B	CR; continued to ASCT—NED since
**9**	0	1	66	Mantle cell	de novo	0	3	No	RDHAOx	C	CR; continued to ASCT—NED since
**10**	0	1	56	DLBCL	relapsed	0	4	Yes	RDHAOx	C	PR -> Rituximab polatuzumab + CAR-T

AE—adverse event; AITL—angioimmunoblastic T cell lymphoma; ASCT—high-dose chemotherapy with stem cell support (autologous bone marrow transplant); BM—bone marrow; CAR-T—chimeric antigen receptor-modified T cell therapy; CR—complete response; DLBCL—diffuse large B cell lymphoma; NED—no evidence of disease; PLT—platelet; PR—partial response; [RCHOP]-RICE—first-line regimen of [rituximab cyclophosphamide doxorubicin vincristine and prednisone] followed by rituximab ifosfamide carboplatin and etoposide, where the qualifying regimen was RICE; RDHAOx—rituximab dexamethasone high-dose cytarabine and oxaliplatin; RGemOx—rituximab gemcitabine and oxaliplatin.

**Table 2:** Outcomes.

	Cohort A (9 Cycles/5 pts)	Cohort B + C (8 Cycles/4 pts)	Total (17 Cycles/9 pts)
sCIT/transfusion * prior to enrollment	5/5 (100%)	4/4 (100%)	9/9 (100%)
Recurrent sCIT	7/9 (78%)	2/8 (25%)	9/17 (53%)
Platelet Transfusions	5/9 (56%)	1/8 (13%)	6/17 (35%)

* All patients experienced sCIT and required at least 1 platelet transfusion in their most recent chemotherapy cycle prior to enrollment. pts—patients; sCIT—severe chemotherapy-induced thrombocytopenia.

## Data Availability

Data is unavailable due to privacy restrictions.

## Supplementary Materials

The following supporting information can be downloaded at: https://www.mdpi.com/article/10.3390/cancers18020188/s1, Table S1. Platelet parameters during the qualifying treatment cycle (before enrollment).

## Abbreviations

The following abbreviations are used in this manuscript:

AE	Adverse Event
AITL	Angioimmunoblastic T Cell Lymphoma
ASCT	High-Dose Chemotherapy with Stem Cell Support (Autologous Bone Marrow Transplant)
BM	Bone Marrow
C1D1	Cycle 1 Day 1
C2D1	Cycle 2 Day 1
C2D8	Cycle 2 Day 8
C2D14	Cycle 2 Day 14
C2D15	Cycle 2 Day 15
CAR-T	Chimeric Antigen Receptor Modified T Cell Therapy
CNS	Central Nervous System
CR	Complete Response
CTCAE	Common Terminology Criteria for Adverse Events
DLBCL	Diffuse Large B Cell Lymphoma
G-CSF	Granulocyte Colony-Stimulating Factor(s)
HD	Hodgkin’s Disease/Hodgkin’s Lymphoma
IRB	Institutional Review Board
ITP	Immune Thrombocytopenic Purpura
MCL	Mantle Cell Lymphoma
NCCN	National Comprehensive Cancer Network
NED	No Evidence of Disease
PEG-rHuMGDF	Pegylated Recombinant Human Megakaryocyte Growth and Development Factor
PLT	Platelet/Platelet Count
PR	Partial Response
R/R	Relapsed or Refractory
RCHOP	Rituximab Cyclophosphamide Doxorubicin Vincristine and Prednisone
RDHAOx	Rituximab Dexamethasone High-Dose Cytarabine and Oxaliplatin
RGemOx	Rituximab Gemcitabine and Oxaliplatin
RICE	Rituximab Ifosfamide Carboplatin and Etoposide
sCIT	Severe Chemotherapy-Induced Thrombocytopenia
TPO-RA	Thrombopoietin Receptor Agonist(s)
