# Supplementary material for: Romiplostim for Prevention of Severe Chemotherapy-Induced Thrombocytopenia in Lymphoma Patients—Phase I Study
Source: Cancers (Basel). 2026 Jan 6;18(2):188. doi: 10.3390/cancers18020188 (PMC12839400; doi:10.3390/cancers18020188)
Supplement: Supplementary file 1 [file cancers-18-00188-s001.zip › cancers-4041875-supplementary.pdf]

Supplementary Table S1. Platelet parameters during the qualifying treatment cycle (before enrollment).

ID	Dose Delay	PLT <50/mcL (days)	PLT Transfusion
1	0	9	2
2	0	7	1
4	0	9	2
5	0	10	2
6	0	11	1
7	0	5	2
8	0	7	1
9	0	5	1
10	0	8	1
